# Associations between Dairy Intake, Body Composition, and Cardiometabolic Risk Factors in Spanish Schoolchildren: The Cuenca Study

**DOI:** 10.3390/nu11122940

**Published:** 2019-12-03

**Authors:** Noelia Lahoz-García, Marta Milla-Tobarra, Antonio García-Hermoso, Monserrat Hernández-Luengo, Diana P. Pozuelo-Carrascosa, Vicente Martínez-Vizcaíno

**Affiliations:** 1 Centro de Estudios Socio-Sanitarios, Universidad de Castilla-La Mancha, 16071 Cuenca, Spain; noelia.lahoz@alu.uclm.es (N.L.-G.); mmillatobarra@gmail.com (M.M.-T.); monserrath@sescam.jccm.es (M.H.-L.); Vicente.martinez@uclm.es (V.M.-V.); 2 Departamento de Nutrición y Dietética, SESCAM, Talavera de la Reina, 45600 Toledo, Spain; 3 Laboratorio de Ciencias de la Actividad Física, el Deporte y la Salud, Facultad de Ciencias Médicas, Universidad de Santiago de Chile, USACH, Santiago 7500618, Chile; antonio.garcia.h@usach.cl; 4 Navarrabiomed, Complejo Hospitalario de Navarra (CHN)-Universidad Pública de Navarra (UPNA), IdiSNA, 31008 Pamplona, Spain; 5 Gerencia de Atención Integrada de Cuenca, SESCAM, 16002 Cuenca, Spain; 6 Facultad de Ciencias de la Salud, Universidad Autónoma de Chile, Talca 1670, Chile

**Keywords:** schoolchildren, obesity, lipid profile, food frequency questionnaire, dairy intake, cardiorespiratory fitness

## Abstract

Full-fat dairy has been traditionally associated with obesity and cardiovascular disease (CVD); however, recent evidence shows that the amount of dairy intake might have a beneficial effect over these pathologies, regardless of their fat content. The aim of this study was to examine the association between the intake of dairy products (including milk with different fat contents) with both adiposity and serum lipid concentration, adjusted by cardiorespiratory fitness (CRF), in Spanish schoolchildren. A cross-sectional study of 1088 children, aged 8 to 11 years, was conducted in which anthropometric variables (body mass index (BMI), waist circumference (WC), fat mass percentage (FM%) and fat mass index (FMI)), blood lipid profile, and dairy intake (using a food frequency questionnaire), and CRF (through a 20-m shuttle run test) were measured. Results showed that children with lower BMI, WC, FM%, and FMI had higher whole-fat milk intake and lower skimmed and semi-skimmed milk intake than children with higher BMI, WC, FM%, and FMI. Children with normal levels of triglycerides and high density lipoproteins (HLD) cholesterol consumed more whole-fat milk and less reduced-fat milk than children with dyslipidemic patterns. These relationships persisted after adjustment for CRF. Our findings suggest that full-fat milk intake should be promoted in children without obesity or high cardiometabolic risk.

## 1. Introduction

In the last decades, the prevalence of childhood obesity has increased in developed and developing countries [1], which poses a serious global public health problem [1,2]. In Spain, more than one out of three children are overweight or obese [3]. Obesity has been associated with short- and long-term complications [2,4], such as increased risk for cardiovascular diseases (CVD), which makes the identification of risk factors related to obesity in the early stages of life crucial.

There is consistent evidence of the association between diet and physical activity and obesity [5] since they are the two pillars of the energy balance. Dietary recommendations state that dairy is a key element of a healthy eating pattern [6], with specific benefits for growth and bone health during childhood [7]. Additionally, some reviews have showed the protective effect of dairy against obesity [8,9] and several studies have found that dairy intake decreases the risk of CVD [10,11] in children and adolescents. However, full-fat dairy products have been traditionally associated with obesity and adverse cardiovascular health due to their fat content and the presence of saturated fatty acid (SFA) in adults [12]. This could be one of the reasons, along with the substitution of milk with other beverages [7,13] or less heathy foods like refined carbohydrates [11], why dairy consumption has decreased in recent decades [9,13]. Even though dairy intake has decreased, even below the national recommendations [6,14], the prevalence of obesity among children has increased [9]. Some studies have attempted to identify if regular and reduced-fat dairy have different effects over CVD in adolescents and failed [11]. Thus, despite the published evidence, actual recommendations have not yet suggested a greater consumption of full-fat dairy instead of low-fat or non-fat dairy products [15], and more data are necessary to corroborate this. Since cardiorespiratory fitness (CRF) has been related to cardiometabolic risk [16,17] and dairy pattern consumption [18,19] in children and adolescents, this study aimed to explore the association between dairy intake (including whole-fat milk, (semi-) skimmed milk, cheese, and other milk products) and both adiposity and serum lipid profiles in Spanish schoolchildren, controlling for CRF, something that, as far as we know, has not been previously reported.

## 2. Materials and Methods

### 2.1. Design

The baseline measurements of a cluster-randomized trial that involved 20 schools in which all the 4th and 5th children were invited to participate, were used for this cross-sectional analysis. In the schools belonging to the intervention group, the Movi-2 program was implemented (register at Clinicaltrials.gov, NCT01277224). This program consisted of 330 min/week in three sessions of recreational physical activity in after-school time during one academic year. The study protocol was approved by the Clinical Research Ethics Committee of the “Virgen de la Luz” Hospital in Cuenca. Parents of participating children provided written consent. A total of 1088 schoolchildren participated, of the 1592 invited, who were 8 to 11 years of age, from 20 schools in the province of Cuenca, Spain. There were no differences in the socio-demographic characteristics between children who participated in our study and eligible children who did not. The selection of schools, the data collection procedure, and the cluster-randomized trial objective have been previously described [20].

### 2.2. Measurement

#### 2.2.1. Anthropometrics

The children were weighed twice whilst wearing light clothing and without shoes using a digital scale to the nearest 0.1 kg (Seca 861). Height was measured using a wall-mounted stadiometer (Seca 222) to the nearest 0.1 cm. The body mass index (BMI) was calculated as weight/height^2^ (kg/m^2^). The waist circumference (WC) was measured with flexible tape in the midpoint between the iliac crest and the last rib and expressed in cm. The fat mass (FM%) percentage was estimated using a bioelectrical impedance analysis system (BC-418, Tanita Corp, Tokyo, Japan). The Tanita BC-418 single frequency model has showed a strong linear correlation with the gold standard method dual energy X-ray absorptiometry (DXA) in 7- to 12-year-old children, although it underestimates fat mass and fat percentage [21]. The fat mass index (FMI = fat mass/height^2^—in kg/m^2^) was calculated. Since weight status is the consequence of an excess of fat and/or lean mass, this index allows the estimation of an indicator of adiposity adjusted by stature [22].

All physical examinations were performed in the morning in schools by trained nurses.

#### 2.2.2. Diet

Dairy habitual consumption was assed using the Health Behavior in School-aged Children (HBSC) food-frequency questionnaire (FFQ) completed by parents. Dairy foods groups selected were whole-fat milk, skimmed and/or semi skimmed milk, cheese, and other milk products (which included yoghurt, chocolate milk, puddings, etc.). Skimmed and semi-skimmed milk were measured together since they provide the same nutrients and similar fat [6]. Dairy consumption was calculated as the sum of these four foods groups. Other food group like fruits, vegetables, cereals, white bread, brown bread, carbonated sugared soft drinks, diet soft drinks, crisps, chips, sweets, and chocolates were also assed. For each item, the questionnaire offered seven frequency response options, from “never” to “more than once a day”. The FFQ allows ranking of the diet of an individual, and its relative validity has been proven [23].

### 2.3. Biochemical Assessments

Blood samples were obtained in standardized conditions between 8:15 and 9:00 a.m. by puncturing the cubital vein. Before the extraction, the child and his parents confirmed at least 12 h of fasting. The samples were processed using a Roche Diagnostics COBAS C711. The following parameters were determined: Triglyceride (glycerolphosphate oxidase, peroxidase enzymatic method), total cholesterol, high density lipoproteins cholesterol (HDL-c), and low density lipoproteins cholesterol (LDL-c) (MODULAR DPP system).

### 2.4. Cardiorespiratory Fitness

CRF was assessed by the 20-m shuttle run test [24]. In this test, the participants were encouraged to run between two lines, 20 m apart, while keeping pace with audio signals emitted from a pre-recorded compact disc. Speed was increased from 8.5 kmh^−1^ by 0.5 kmh^−1^ min^−1^ (stage duration = 1 min). The test was completed when the child failed to reach the end line at the same time indicated by the audio signal. The last half stage completed by the child was noted as an indicator of his/her CRF.

### 2.5. Statistical Analysis

The distribution of continuous variables was checked for normality. Descriptive statistics, including means and standard deviation (SD) for continuous variables, and n and percentage (%) for categorical variables, were calculated. Differences among sexes in continuous variables were examined by Student’s t test for independent samples and Pearson’s χ^2^ for categorical variables. There were no differences in the socio-demographic characteristics between children who participated in our study and eligible children who did not.

Correlation coefficients were estimated to examine the relation of food group intake measured with adiposity variables, serum lipid concentration, and CRF.

To improve the handling of food intake frequencies, the FFQ response options were recoded to represent the average weekly consumption as follows: “Never” = 0; “less than once a week” = 0.25 (estimating a consumption frequency of once every month); “once a week” = 1; “2–4 days a week” = 3 (center of the interval); “5–6 days a week” = 5.5 (middle of the interval); and “once a day, every day” and “more than once a day, every day” = 7.

Children were classified as normal weight, overweight, or obese according to gender- and age-specific BMI cut-offs defined by Cole et al. [25]. We categorized WC, FM%, and FMI as low (first quartile), medium (second and third quartiles), or high (fourth quartile). The variables BMI categories, WC, FM%, and FMI were used as indicators of adiposity.

Adverse lipid concentrations in children were defined as follows: Total cholesterol concentrations ≥200 mg/dL; HDL-c < 40 mg/dL; LDL-c concentrations ≥130 mg/dL; and triglyceride concentrations ≥100 mg/dL for children under 10 years and ≥130 mg/dL for children over 10 [26].

Differences in the means of the food group intake between categories of BMI, WC, FM%, FMI, and categories of cardiometabolic risk factors (non-risk vs. at-risk children) were done with one-way analyses of covariance (ANCOVA). The analyses were controlled for sex and age (model 1) and with further adjustment for CRF (model 2). Pairwise comparisons were conducted by Bonferroni post-hoc test.

A bilateral criterion for statistical significance of *p* ≤ 0.05 was used. All statistical analyses were performed using the software IBM SPSS 23 (SPSS, Inc., Chicago, IL, USA).

## 3. Results

### 3.1. Descriptive Statistics

Descriptive information of the participants of the study is presented in Table 1. Boys showed higher WC, CRF, and HDL-c than girls; however, girls showed higher FM%, FMI, and triglycerides than boys. Regarding the dairy food groups, only “other milk products” intake showed differences among sexes.

Table 2 presents the correlation between adiposity variables, serum lipid concentrations, and CRF, with the dairy food group intakes measured. All the variables related to adiposity; moreover, triglycerides and HDL-c showed a correlation with whole-fat milk and (semi-) skimmed milk. LDL-c indicated a correlation with whole-fat milk. 

### 3.2. Dairy Intake and Adiposity

Table 3 shows the differences in weekly intake of food groups by BMI, WC, FM%, and FMI categories. Whole-fat milk intake decreases while BMI, WC, FM%, and FMI increase. In contrast, (semi-) skimmed milk intake increases while BMI, WC, FM%, and FMI increase.

### 3.3. Dairy Intake and Serum Lipid Profile

Table 4 shows differences in dairy food groups between categories of cardiometabolic risk (non-risk vs. at-risk children). A higher intake of whole-fat milk and lower intake of (semi-) skimmed milk was found in children with better levels of triglycerides and HDL-c.

When CRF was added to the analysis as a covariate (model 2), most of these results remained significant. Carbonated drink intake was added as a covariable since its consumption had increased concurrently with a decrease in milk intake [9], but the results did not show significant changes (data not showed).

## 4. Discussion

### 4.1. Statement of Principal Findings

This study aimed to examine the differences in milk and dairy product consumption by categories of adiposity and cardiometabolic risk in a sample of Spanish schoolchildren. Overall, our results showed an inverse relationship between whole-fat milk consumption and cardiometabolic risk, as expressed by adiposity measures and lipid profiles and a positive relationship between skimmed and semi-skimmed milk consumption and cardiometabolic risk variables.

### 4.2. Direction of the Association between Dairy Intake and Adiposity

We found an inverse association between whole-fat milk intake and several measurements of adiposity. The relationship between milk or dairy intake and obesity in children had been previously examined in several studies with similar results [10,27,28]. Existing literature showed consistently that dairy and obesity in children were either inverse or not associated [8]. In consequence, published reviews indicated that the protective effect of dairy intake on obesity and overweight is only suggestive [8,9,29,30]. The high study heterogeneity in the measurement of dairy (type of dairy assessed, differences in composition and portions) as well as the variable used to evaluate obesity could be responsible for this [8]. We also found a positive association between (semi-) skimmed milk consumption and FM%, confirming previous results from the National Health and Nutrition Examination Survey (NHANES) III study [27]. Since total energy intake is similar in children with regular or reduced-fat dairy intake, our results could be explained as the consumption of reduced fat dairy seems to be linked to refined carbohydrates’ intake [11]. That means dairy (a nutrient-dense food) is replaced by a less healthy energy-dense choice [7]. Another possible explanation could be based on the long-term effect of early nutrition since low-fat intake is associated with higher trunk body fat and development of leptin resistance in adulthood [31]. In addition, low-fat dairy contains higher energy from proteins than whole-fat dairy, which has been associated with other long-term effects, such as earlier adiposity rebound in children and obesity in adulthood [32].

When we accumulated the various types of milk, cheese, and other milk products, such as dairy, we did not find differences in consumption based on BMI, WC, FM%, or FMI. In contrast, previous studies have shown a relationship between dairy and obesity in children and adolescents [27,33]. However, one meta-analysis that included 22 studies did not find any statistically significant association between dairy intake and adiposity in the aggregated data [9]; only when the sample was divided into two different age groups (2–11 vs. ≥12) was an association found [9]. The slight relationship between dairy and obesity in children reported in some reviews [29,30,34] could explain the absence of an association that we observed in our data.

### 4.3. Direction of the Association between Dairy Intake and Serum Lipid Profile

We found that whole-fat milk intake is negatively associated with triglyceride concentration while the opposite occurred with reduced-fat milk intake. In contrast, HDL-c is positively associated with full-fat milk intake while HDL-c is negatively associated with (semi-) skimmed milk intake. Our results showed that milk fat content does not seem to affect total cholesterol or LDL-c. There is little evidence examining the associations between dairy intake, particularly regular compared with reduced-fat dairy, and cardiometabolic risk factors in children or adolescence. Previous experimental research in young men revealed that two diets based on milk and cheese, respectively, did not increase total and LDL-c concentrations as much as a control diet with the same content of SFAs without dairy products [12]. When comparing dairy intake, we did not find differences based on triglycerides, total cholesterol, or LDL-c levels. These results are in line with a systematic review of observational studies in children and adults that concluded that dairy intake did not contribute to CVD risk, in spite of its high SFA content [35]. Most of these results are based on total dairy consumption, including full-fat and reduced-fat versions, which could debunk the attributed negative effects of full fat dairy over obesity. It seems that the type of fat and other components of dairy should have a beneficial effect over obesity and CVD risk. Likewise, the essential fatty acid linoleic, found in whole-fat dairy, has confirmed an inverse association with obesity [35] and has shown a small effect or no effect on lipid concentration [35]. Thereby, some studies have found an inverse association between calcium intake and FM% in children [36] and between calcium intake and BMI and WC in female adolescents [37]. Clinical trials have confirmed the lipolytic effect of calcium intake through various processes [12,34,38]. Some of the effects observed are greater when analyzing dairy consumption instead of calcium intake alone due to other bioactive compounds that reinforce these mechanisms [34].

According to national dietary guidelines, children must consume between two and four servings of milk or dairy per day [14], similar to the Dietary Guidelines for Americans, which recommend 2.5 to 3 cups of milk products in this age group [6]. However, our data showed that for all the adiposity and lipid serum categories, the average intake of dairy was slightly below the recommendation. Similar results were found in American children [6]. These results suggest that not all children meet the dietary recommendation for dairy, and due to the possible protective effect of milk and dairy consumption on obesity, this could be one of the causes of the obesity epidemic in children. This reinforced the suggestions of other authors [9]. Based on our results and the existing literature, we were not able to elucidate if the presence or absence of fat in milk and dairy could produce better health results. Therefore, the advantage of high-fat dairy over low-fat or non-fat dairy products on obesity cannot be established [15,35]. However, more experimental studies are necessary since consumption of fat-reduced dairy by children would diminish essential fatty acid and fat-soluble vitamin intake.

### 4.4. Limitations

The current study has several limitations. First, the cross-sectional design did not allow for causal relationships between variables to be established. Second, the HBSC FFQ was reported by parents, and children might have consumed dairy unbeknownst to their parents, which could favor underreporting. However, the FFQ used in this study showed that it had sufficient reliability and validity to rank subjects according to food group consumption when compared with a 24-h food behavior checklist and a 7-day food diary [23]. Thirdly, some analyzed food groups were very heterogeneous since we did not control for calcium-enriched milk intake or the fat content of different types of cheese; however, our objective was not to determine dietary intake in children but only to rank their dairy consumption. We have previously shown an inverse association between fat intake (through two 24-h dietary recalls) and obesity [39]. Furthermore, no data of portion sizes were gathered. Nevertheless, we thought that portion sizes did not vary too much among parents in our sample since they came from a “small” territory with a similar culture. Finally, we did not know if children were breastfeeding or complementary feeding during the first stages of life and adjusting for this could be necessary due to the different fat and protein contents of both milks and the long-term effects of that nutritional intake on obesity [31,32].

## 5. Conclusions

Findings from our study revealed that high-fat and fat-free plus low-fat milk consumption varies according to adiposity and lipid profile in 8- to 11-year-old schoolchildren. Despite the cross-sectional design of the present study, the results seem to reinforce the importance of promoting full-fat dairy products in a normal healthy diet due to the potential benefits of essential fatty acids and other components, with no adverse effect on obesity or CDV risk.

## Figures and Tables

**Table 1:** Characteristics of the study sample by sex.

	Total (*n* = 1088)	Boys (*n* = 548)	Girls (*n* = 540)
Age	9.5 ± 0.7	9.5 ± 0.7	9.5 ± 0.7
Weight, kg	37.4 ± 9.2	37.7 ± 9.6	37.0 ± 8.8
Height, m	139.6 ± 7.0	139.8 ± 7.3	139.4 ± 6.8
BMI, kg/m^2^	19.0 ± 3.7	19.2 ± 3.8	18.8 ± 3.5
Normal weight ^1^	702 (64.5)	342 (62.4)	360 (66.7)
Overweight	277 (25.5)	148 (27.0)	129 (23.8)
Obese	109 (10.0)	58 (10.6)	51 (9.5)
Waist circumference, cm	67.6 ± 9.4	68.2 ± 9.7 *	67.0 ± 9.0 *
FM%	25.4 ± 6.8	23.9 ± 7.1 **	26.8 ± 6.1 **
FMI (kg/m^2^)	5.0 ± 2.3	4.8 ± 2.5 *	5.2 ± 2.2 *
Triglycerides, mg/dL	68.7 ± 34.4	65.2 ± 34.8 **	72.2 ± 36.2 **
Total cholesterol, mg/dL	169.0 ± 27.6	169.3 ± 27.0	168.8 ± 28.1
HDL cholesterol, mg/dL	59.6 ± 13.3	61.1 ± 13.8 **	58.1 ± 12.6 **
LDL cholesterol, mg/dL	97.2 ± 23.3	96.6 ± 22.7	97.8 ± 23.8
CRF Stage, *n*	3.5 ± 1.7	4.1 ± 1.8 **	2.9 ± 1.3 **
Weekly consumption			
Whole fat milk	3.5 ± 3.4	3.5 ± 3.4	3.4 ± 3.4
(Semi-) skimmed milk	3.7 ± 3.4	3.7 ± 3.4	3.8 ± 3.4
Cheese	2.8 ± 2.3	2.8 ± 2.3	2.8 ± 2.4
Other milk products	4.6 ± 2.4	4.8 ± 2.4 *	4.4 ± 2.5 *
Total dairy products	12.8 ± 5.9	12.7 ± 6.1	12.9 ± 5.7

BMI: body mass index; FM%: fat mass percentage; FMI: fat mass index; HDL: high density lipoprotein; LDL: low density lipoprotein; CRF: cardiorespiratory fitness; ^1^ Gender- and age-specific BMI cut off defined by Cole et al. [25]. Data are presented as mean ± S.D and number and proportions (%) for categorical data. Gender group comparisons were conducted by Student’s t-test for continuous variables and Pearson’s χ^2^ for categorical variables. * *p* < 0.05; ** *p* < 0.001.

**Table 2:** Correlation coefficients between food group consumption with cardiometabolic risk variables and cardiorespiratory fitness.

Variables	Whole Fat Milk	(Semi-) Skimmed Milk	Cheese	Other Milk Products	Total Dairy Products
BMI	−0.238 **	0.216 **	0.047	−0.045	−0.046
WC	−0.205 **	0.181 **	0.074 *	−0.017	−0.033
FM%	−0.213 **	0.189 **	0.045	−0.072 *	−0.012
FMI	−0.238 **	0.210 **	0.041	−0.061 *	−0.034
Triglycerides	−0.108 **	0.106 **	0.042	−0.047	−0.051
Total cholesterol	−0.058	−0.001	0.081 *	−0.022	0.024
HDL cholesterol	0.085 **	−0.128 **	0.028	0.050	0.069 *
LDL cholesterol	−0.090 **	0.048	0.065 *	−0.042	−0.007
CRF	0.124 **	−0.089 **	−0.045	0.065 *	−0.002

BMI: body mass index; WC: waist circumference; FM%: fat mass percentage; FMI: fat mass index; HDL: high density lipoprotein; LDL: low density lipoprotein; CRF: cardiorespiratory fitness. * *p* < 0.05; ** *p* < 0.001.

**Table 3:** Differences in weekly dairy consumption by adiposity categories.

		Model 1					Model 2				
		Whole fat milk	(Semi-) skimmed milk	Cheese	Other milk products	Total dairy products	Whole fat milk	(Semi-) skimmed milk	Cheese	Other milk products	Total dairy products
BMI ^1^	Normal weight (*n* = 698)	4.0 ± 0.1 ^a,b^	3.3 ± 0.1 ^a,b^	2.8 ± 0.1	4.7 ± 0.1	13.0 ± 0.2	4.0 ± 0.1 ^a,b^	3.4 ± 0.1 ^a,b^	2.8 ± 0.1	4.7 ± 0.1	13.0 ± 0.2
	Overweight (*n* = 279)	2.9 ± 0.2 ^c^	4.1 ± 0.2 ^c^	2.9 ± 0.1	4.6 ± 0.1	12.8 ± 0.3	2.9 ± 0.2 ^c^	4.2 ± 0.2 ^c^	2.8 ± 0.2	4.7 ± 0.2	12.8 ± 0.4
	Obese (*n* = 111)	1.4 ± 0.4	5.5 ± 0.3	3.0 ± 0.2	4.4 ± 0.2	11.6 ± 0.5	1.5 ± 0.4	5.5 ± 0.4	2.9 ± 0.3	4.4 ± 0.3	11.6 ± 0.6
	*p*	**<0.001**	**<0.001**	0.770	0.491	0.075	**<0.001**	**<0.001**	0.938	0.665	0.114
WC	Low (*n* = 281)	4.0 ± 0.2 ^e^	3.2 ± 0.2 ^e^	2.4 ± 0.1 ^d,e^	4.7 ± 0.1	12.8 ± 0.3	3.9 ± 0.2 ^e^	3.4 ± 0.2 ^e^	2.4 ± 0.2 ^d^	4.6 ± 0.2	12.7 ± 0.4
	Medium (*n* = 535)	3.7 ± 0.2 ^f^	3.6 ± 0.2 ^f^	3.0 ± 0.1	4.6 ± 0.1	12.9 ± 0.2	3.7 ± 0.2 ^f^	3.6 ± 0.2 ^f^	3.0 ± 0.1	4.6 ± 0.1	12.9 ± 0.3
	High (*n* = 271)	2.3 ± 0.2	4.7 ± 0.2	3.0 ± 0.1	4.6 ± 0.2	12.6 ± 0.3	2.4 ± 0.2	4.7 ± 0.2	3.0 ± 0.2	4.7 ± 0.2	12.8 ± 0.4
	*p*	**<0.001**	**<0.001**	**0.005**	0.906	0.850	**<0.001**	**<0.001**	**0.009**	0.914	0.862
FM%	Low (*n* = 271)	4.2 ± 0.2 ^e^	3.0 ± 0.2 ^e^	2.5 ± 0.2	4.8 ± 0.2	12.5 ± 0.4	4.2 ± 0.2 ^e^	3.1 ± 0.2 ^e^	2.5 ± 0.2	4.8 ± 0.2	12.4 ± 0.4
	Medium (*n* = 543)	3.7 ± 0.2 ^f^	3.6 ± 0.1 ^f^	2.9 ± 0.1	4.7 ± 0.1	13.1 ± 0.2	3.7 ± 0.2 ^f^	3.7 ± 0.2 ^f^	2.9 ± 0.1	4.7 ± 0.1	13.2 ± 0.3
	High (*n* = 274)	2.3 ± 0.2	4.7 ± 0.2	2.9 ± 0.1	4.4 ± 0.2	12.4 ± 0.3	2.3 ± 0.2	4.8 ± 0.2	2.9 ± 0.2	4.3 ± 0.2	12.4 ± 0.4
	*p*	**<0.001**	**<0.001**	0.079	0.130	0.130	**<0.001**	**<0.001**	0.145	0.138	0.072
FMI	Low (*n* = 274)	4.1 ± 0.2 ^e^	3.1 ± 0.2 ^e^	2.6 ± 0.1	4.8 ± 0.2	12.6 ± 0.3	4.0 ± 0.2 ^e^	3.1 ± 0.2 ^e^	2.5 ± 0.2	4.9 ± 0.2	12.5 ± 0.4
	Medium (*n* = 542)	3.7 ± 0.1 ^f^	3.6 ± 0.1 ^f^	2.9 ± 0.1	4.6 ± 0.1	13.0 ± 0.2	3.7 ± 0.2 ^f^	3.6 ± 0.2 ^f^	2.9 ± 0.1	4.6 ± 0.1	13.1 ± 0.3
	High (*n* = 272)	2.3 ± 0.2	4.7 ± 0.2	2.9 ± 0.1	4.4 ± 0.2	12.5 ± 0.3	2.4 ± 0.2	4.9 ± 0.2	2.9 ± 0.2	4.4 ± 0.2	12.6 ± 0.4
	*p*	**<0.001**	**<0.001**	0.081	0.191	0.360	**<0.001**	**<0.001**	0.167	0.214	0.246

Data are presented as marginal estimated mean ± S.E. ANCOVA model 1: controlling for age and sex. Model 2 further adjustments for cardiorespiratory fitness. Abbreviations: BMI: body mass index; WC: waist circumference; FM%: fat mass percentage; FMI: fat mass index. ^1^ Gender- and age-specific BMI cut off defined by Cole et al. [25]. ^a^ Significant difference between normal weight and overweight; ^b^ Significant difference between normal weight and obese; ^c^ Significant difference between overweight and obese. ^d^ Significant difference between low and medium; ^e^ Significant difference between low and high; ^f^ Significant difference between medium and high. All the pairwise mean comparisons using Bonferroni post hoc test were statistically significant as shown in boldface type (*p* < 0.05).

**Table 4:** Differences in weekly dairy consumption by serum lipid concentration risk category.

		Model 1					Model 2				
		Whole fat milk	(Semi-) skimmed milk	Cheese	Other milk products	Total dairy products	Whole fat milk	(Semi-) skimmed milk	Cheese	Other milk products	Total dairy products
Triglycerides	Normal (*n* = 980)	3.5 ± 0.1	3.7 ± 0.1	2.8 ± 0.1	4.6 ± 0.1	12.9 ± 0.2	3.5 ± 0.1	3.7 ± 0.1	2.8 ± 0.1	4.6 ± 0.1	12.9 ± 0.2
	High (*n* = 108)	2.8 ± 0.4	4.7 ± 0.3	3.2 ± 0.2	4.5 ± 0.2	12.7 ± 0.6	2.9 ± 0.4	4.6 ± 0.4	3.1 ± 0.2	4.6 ± 0.2	12.7 ± 0.6
	*p*	**0.048**	**0.006**	0.177	0.678	0.708	0.134	**0.023**	0.286	0.786	0.773
Total cholesterol	Normal (*n* = 942)	3.5 ± 0.1	3.8 ± 0.1	2.8 ± 0.1	4.7 ± 0.1	12.9 ± 0.2	3.5 ± 0.1	3.8 ± 0.1	2.4 ± 0.2	4.7 ± 0.1	12.9 ± 0.2
	High (*n* = 146)	3.1 ± 0.3	3.8 ± 0.3	2.9 ± 0.2	4.6 ± 0.2	12.8 ± 0.5	3.2 ± 0.2	3.8 ± 0.3	3.2 ± 0.2	4.6 ± 0.2	12.9 ± 0.5
	*p*	0.192	0.853	0.624	0.669	0.925	0.312	0.924	0.803	0.600	0.873
HDL cholesterol	Normal (*n* = 1038)	3.5 ± 0.1	3.7 ± 0.1	2.8 ± 0.1	4.6 ± 0.2	12.9 ± 0.2	3.5 ± 0.1	3.7 ± 0.1	2.8 ± 0.1	4.6 ± 0.1	12.9 ± 0.2
	Low (*n* = 50)	2.0 ± 0.5	5.4 ± 0.5	3.1 ± 0.4	4.7 ± 0.2	12.7 ± 0.8	2.0 ± 0.5	5.5 ± 0.5	3.1 ± 0.4	4.8 ± 0.4	13.2 ± 0.9
	*p*	**0.004**	**0.001**	0.529	0.879	0.876	**0.011**	**0.002**	0.432	0.579	0.705
LDL cholesterol	Normal (*n* = 9 99)	3.5 ± 0.1	3.7 ± 0.1	2.9 ± 0.1	4.7 ± 0.1	12.9 ± 0.2	3.5 ± 0.1	3.8 ± 0.1	2.8 ± 0.1	4.7 ± 0.1	12.8 ± 0.2
	High (*n* = 89)	3.2 ± 0.4	4.0 ± 0.4	2.9 ± 0.3	4.5 ± 0.3	12.8 ± 0.6	3.3 ± 0.4	4.0 ± 0.4	2.8 ± 0.3	4.4 ± 0.3	13.2 ± 0.6
	*p*	0.449	0.554	0.851	0.476	0.918	0. 696	0.617	0.926	0.423	0.631

Data are presented as marginal estimated mean ± S.E. ANCOVA model 1: controlling for age and sex. Model 2: further adjustments for cardiorespiratory fitness. Abbreviations: HDL: high density lipoprotein; LDL: low density lipoprotein. Adverse serum lipid concentrations were defined as follows: triglycerides concentrations ≥ 100 mg/dL for children under ten years and ≥ 130 mg/dL for children over ten; total cholesterol concentrations ≥ 200 mg/dL; HDL cholesterol concentrations < 40 mg/dL and LDL cholesterol ≥ 130 mg/dL [26]. All the pairwise mean comparisons using Bonferroni post hoc test were statistically significant as shown in boldface type (*p* < 0.05).
